# PREDICTING FALLS IN PARKINSON'S DISEASE

**DOI:** 10.1093/geroni/igz038.3332

**Published:** 2019-11-08

**Authors:** Ingrid A Pretzer-Aboff, and Ronald K Elswick

**Affiliations:** Virginia Commonwealth University School of Nursing, Richmond, Virginia, United States

## Abstract

People with Parkinson’s disease (PD) have a proclivity to falling. Early identification and treatment of PD patients with a high risk of falling is important to decrease morbidity, mortality, and improve quality of life. We compared functional performance tests and balance tests in people with PD to determine which tests predict fallers. Sixty participants were recruited, mean age 71.4 y.o. (range 55 – 89), 43 were male, and 34 were identified as fallers (defined as having fallen at least twice in the past year). A logistic regression model was built to determine which functional performance and balance tests would best predict fallers. Predictors in the model included Hoehn and Yahr stage of disease, Unified Parkinson Disease Rating Scale (UPDRS), Barthel Index, Timed Up and Go (TUG), Tinetti Balance Assessment, Berg Balance Scale, and results from a force plate that recorded sway in both static and dynamic conditions (open eyes and closed eyes). Correlations among predictor variables were assessed for multicollinearity and were less than 0.8. Using both a forward and backward stepwise approach, the best prediction model included Tinetti Balance Total score only. ROC analysis yielded an area under the curve of 74% with a cutoff of 13 which had a diagnostic accuracy of 68.8% with an 83% specificity and 56% sensitivity. Given that the cost of treatment for an injurious fall far exceeds preventative measures a clinician may opt to use a cut off of 14 when using the Tinetti Balance Assessment given an 70% specificity and 68% sensitivity.

